# Eco-epidemiology of gastrointestinal parasitic infections in captive chimpanzees in Gabon

**DOI:** 10.1016/j.ijppaw.2025.101101

**Published:** 2025-06-10

**Authors:** Mohamed Hassani Mohamed-Djawad, Krista Mapagha-Boundoukou, Neil M. Longo-Pendy, Serge Ely Dibakou, Barthelemy Ngoubangoye, Papa Ibnou Ndiaye, Larson Boundenga

**Affiliations:** a Unité de Recherche en Écologie de la Santé (URES), Centre Interdisciplinaire de Recherches Médicales de Franceville (CIRMF), BP 769, Franceville, Gabon; b Laboratoire de Biologie évolutive Écologie et Gestion des écosystèmes, Département de Biologie animale, Fa-culté des Sciences et Techniques, Université Cheikh Anta Diop, Dakar, Senegal; c Centre De Primatologie (CDP), Centre Interdisciplinaire de Recherches Médicales de Franceville (CIRMF), BP 769, Franceville, Gabon; d Département d'anthropologie, Université de Durham, South Road, Durham, DH1 3LE, UK

**Keywords:** Chimpanzees, Gastrointestinal parasites, Abiotic variables, Conservation, Gabon

## Abstract

This study investigates the influence of abiotic variables (precipitation, soil surface temperature, and soil water content) and intrinsic factors (sex, age class, and social status) on the occurrence of gastrointestinal parasitic infections in two populations of chimpanzees (*Pan troglodytes*) in Gabon: one in captivity at the Primatology Center of CIRMF (n = 41) and the other in semi-captivity at Lékédi Park (n = 46). A total of 87 fecal samples were analyzed using flotation and sedimentation techniques. Fourteen gastrointestinal parasite taxa were identified, including two novel findings in chimpanzees: *Fasciola hepatica* and *Toxocara* sp. The overall prevalence was 85.4 % at the Primatology Center and 95.7 % at Lékédi Park. Binary logistic regression models revealed significant associations between intrinsic traits and parasite occurrence in captive individuals. Juveniles were at higher risk of *Balantioides coli* infection compared to adults (OR = 7.24; 90 % CI: 2.15–24.3; p = 0.047), while subordinate individuals were less likely to be infected than dominants (OR = 0.08; 90 % CI: 0.02–0.165; p = 0.007). Males were significantly more likely to be infected with strongylid nematodes (Strongylida fam. gen.) than females (OR = 6.58; 90 % CI: 1.90–22.7; p = 0.023). No significant associations were found between intrinsic factors and parasite occurrence in semi-captive individuals. Precipitation was significantly associated with increased infection risks in semi-captive chimpanzees, particularly for *Balantioides coli*, *Entamoeba* sp., *Mammomonogamus* sp., *Strongyloides* sp., and *Trichuris* sp. Conversely, in captive chimpanzees, precipitation was negatively associated with *Entamoeba* sp. and Strongylida fam. gen. Soil surface temperature was inversely correlated with the presence of *Balantioides coli*, *Entamoeba* sp., and *Mammomonogamus* sp. in the semi-captive group. No significant associations were detected between soil water content and parasite occurrence. A Spearman rank correlation analysis revealed a strong positive, though non-significant, relationship between parasite prevalence in soil samples and in captive chimpanzees (ρ = 0.82; p = 0.089).

## Highlights

• First detection of *Fasciola hepatica* and T*oxocara* sp. in chimpanzees expands known host range.
• Parasite assemblages differ by management: semi-captivity shows higher prevalence, captivity broader diversity.
• Intrinsic factors (age, sex, dominance) predict infection only in captivity; abiotic drivers dominate in semi-captivity.
• Rainfall boosts soil-borne parasite risk in forest pens but shows a protective effect in concrete enclosures; soil acts as a key environmental reservoir (ρ = 0.82).

## Graphical abstract

**Figure:**
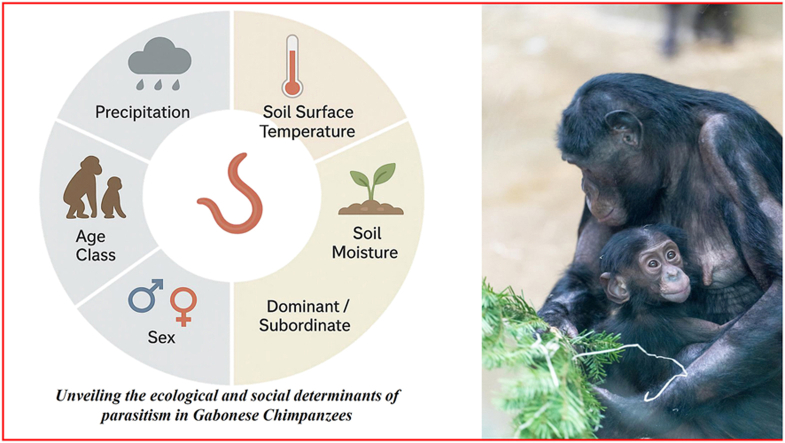

## 1 Introduction

The development of gastrointestinal parasitic infections in non-human primates (NHPs) results from a complex interplay between the host's biological attributes, social dynamics, and the environment. Far from being mere pathogens, these parasites play a fundamental role in regulating populations and maintaining ecosystem balance (Hatcher et al., 2012; Seilacher et al., 2007). Chimpanzees, in particular, are known for their susceptibility to various intestinal parasites (Boundenga et al., 2021; Burgos-Rodriguez, 2011; Friant et al., 2016; Medkour et al., 2020; Ngoubangoye et al., 2021; Willenborg et al., 2022). In captive settings, infectious diseases particularly parasitic infections remain the primary health concern. These conditions create ecological and sanitary contexts that promote increased exposure to gastrointestinal parasites, thereby posing significant challenges for veterinary management (Li et al., 2015; Mbaya and Udendeye, 2011; Ngoubangoye et al., 2021; Willenborg et al., 2022).

Intestinal parasites serve as excellent health indicators (Howells et al., 2011), influencing host population dynamics through disease or reduced reproductive success (Kiene et al., 2021). They also modulate immune responses and, consequently, susceptibility to other pathogens (Halliez and Buret, 2015; Newbold et al., 2017), potentially compromising the nutritional status of the host (Naveed and Abdullah, 2021; Newbold et al., 2017). Moreover, intestinal parasites in NHPs may represent a zoonotic risk to humans, given the close interactions and phylogenetic proximity between the two species (Cibot et al., 2015; Devaux et al., 2019; Gillespie et al., 2008; Köster et al., 2021; Willenborg et al., 2022).

Studies conducted in natural settings have notably identified significant associations between climatic variables and parasite prevalence. For instance, in wild chimpanzees in Uganda, distinct seasonal patterns have been observed, particularly related to rainfall and temperature (Huffman et al., 1997; Krief, 2003; McLennan et al., 2017). Rondón et al. (2017) also noted that a greater parasite diversity can be observed during wetter seasons. However, captive conditions differ significantly from those found in the wild, in terms of population density, resource availability, or care practices, thus limiting the direct transposition of these findings without a specific assessment in a captive context.

Beyond these abiotic factors, individual characteristics also affect parasite prevalence in chimpanzees. Social status, for example, has been correlated with differences in parasitism in various vertebrates (Fugazzola and Stancampiano, 2012; Habig et al., 2019). Additionally, hormonal and immunological differences may explain the higher prevalence or intensity of infection often seen in males (Klein, 2004; Köster et al., 2021). Lastly, young individuals, whose immune systems are still maturing and who frequently engage in social interactions, are often more susceptible to infections (Muehlenbein, 2006; Müller-Klein et al., 2019).

To our knowledge, no study has so far objectively examined the influence of abiotic variables namely precipitation, soil surface temperature, and soil water content as well as intrinsic factors such as sex, age class, and social status on the occurrence of gastrointestinal parasitic infections in chimpanzees kept in captivity or semi-captivity. Addressing this gap is all the more essential, as understanding these parameters is crucial for developing effective veterinary management strategies in confined and high-density environments.

In this context, the present study aims to: (1) determine the diversity, distribution, and prevalence of intestinal parasites in captive and semi-captive chimpanzees, and (2) analyze the influence of abiotic variables (precipitation, soil surface temperature, and soil water content) and individual characteristics (sex, age class, and social status) on the occurrence of gastrointestinal parasitic infections. This approach, grounded in a captive environment, seeks to deepen our understanding of the ecological and physiological drivers of parasite dynamics under controlled conditions and to inform improved veterinary management strategies.

## 2 Methods

### Ethical approval

The study was approved by the National Ethics Committee of Gabon (authorization N°PROT/0024/2018/SG/CNER) and authorized by the Gabonese Ministries of Water and Forests, Higher Education, Scientific Research, and Innovation (Approval No. AR0031/10/MENESRESI/CENAREST/CG/CST/CSAR).

### 2.1 Study sites and population

**Fig. 1:**
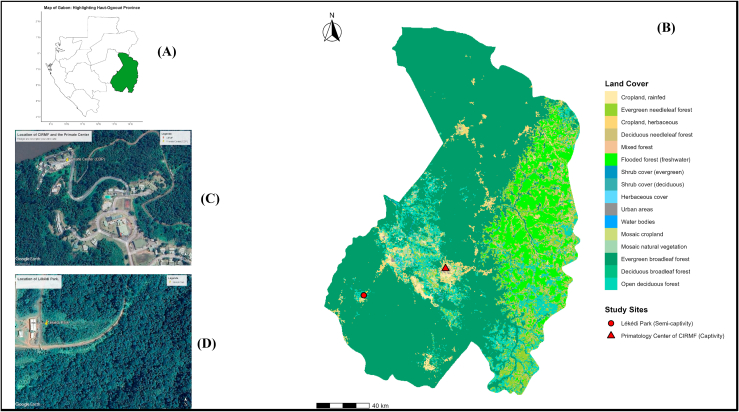
Localization of study site. (A): Map of Gabon with the Haut-Ogooué province highlighted. (B): Detailed view of the Haut-Ogooué province. (C): Aerial view of the Primatology Center of CIRMF. (D): Aerial view of Lékédi Park.

This study was conducted prospectively during both the rainy and dry seasons in the Haut-Ogooué province of Gabon (Fig. 1), focusing on two distinct sites: the Primatology Center of CIRMF (n = 41) and the Lékédi Park (n = 46), for a total of 87 chimpanzees (Table 1). The Primatology Center (CDP), located within the CIRMF campus, spans 49 ha and hosts over 350 primates from ten different species, including approximately forty chimpanzees and 250 mandrills (Unpublished Data) (Fig. 1). At the CDP, chimpanzees are housed in social groups of 5–9 individuals within buildings equipped with aviaries. Each aviary opens onto an enrichment courtyard where the animals spend most of their daytime hours. The enclosures are cleaned every morning by caretakers prior to feeding. On average, the chimpanzees receive a preventive ivermectin-based deworming treatment once per year.

**Table 1:** Demographic distribution of chimpanzees by site, sex, and age class.

Site	Sex	Total (N)	Juveniles (n/N)	Adults (n/N)
**Primatology Center**	Males	25	3/8	22/33
	Females	16	5/8	11/33
**Lékédi Park**	Males	24	12/20	12/26
	Females	22	8/20	14/26

**Note**: *n/N* indicates the number of individuals in each age class (juvenile or adult) out of the total number of individuals by sex.

In contrast, Lékédi Park is a privately-owned, fenced reserve covering 14,000 ha of high biodiversity, and it shelters a semi-captive population of about fifty chimpanzees and approximately 30 gorillas. The chimpanzees at this site live in an environment designed to closely mimic natural conditions, with minimal human interference. There is no systematic antiparasitic treatment protocol in place; rather, deworming is performed only when clinical symptoms of severe parasitic infection are observed and confirmed during veterinary health monitoring.

### 2.2 Sampling, biological materials, and climatic data

Fecal samples were collected non-invasively in accordance with the Pan African Sanctuary Alliance (PASA) recommendations to ensure animal welfare. At both study sites, chimpanzees were identified by their names and distinctive physical traits, facilitating individual tracking. At the Primatology Center, sampling involved isolating individual chimpanzees in compartments specifically designed for that purpose until defecation occurred. Feces were collected immediately after defecation. Approximately 10 g of fecal matter were retrieved using a disposable plastic spoon and placed into sterile containers for coprological analysis. The date, name, and sex of each individual were recorded. Dominant males were identified with the assistance of caretakers, based on observed social behaviors such as priority access to space or displays of strength. Age categories were defined as juveniles (0–12 years) and adults (≥13 years), based on chronological age, physical development, and signs of sexual maturity. Coprological analyses were conducted on average within 1 h of sample collection to ensure optimal preservation of parasite structures.

At Lékédi Park, assistant personnel monitored daily defecation in designated feeding zones. Individuals were recognized by their names and unique traits, and their defecation events were observed in real time to ensure immediate collection. Once defecation was confirmed, samples were promptly collected, and individual data (name, sex, age class, dominance status) were recorded. All chimpanzees at the park are named and easily identifiable by their caretakers.

To investigate parasitic ecology and potential contamination sources, soil samples were collected randomly from the animal enrichment area at the CDP. Approximately 30 g of soil samples were collected at a depth of 5 cm. All samples were air-dried at room temperature for about 24 h and then sieved using a 150 μm filter to remove large particles. A total of 23 soil samples were collected and analyzed.

Climatic data were obtained from the NASA POWER Data Access Viewer (https://power.larc.nasa.gov/data-access-viewer/), which compiles satellite-derived and reanalyzed datasets at various temporal resolutions. For this study, we extracted the monthly averages corresponding to our sampling period in June and October reflecting the dry and rainy seasons in Gabon, respectively. The parameters extracted included surface temperature (TS, °C), reflecting “skin” temperature; surface soil moisture (GWETTOP, unitless), used to assess topsoil water content; and daily precipitation (PRECTOTCORR, mm/day).

### 2.3 Parasite identification

Parasites were detected using two complementary techniques: the McMaster flotation method and the sedimentation technique, following protocols adapted from Pouillevet et al. (2017), using a saturated salt solution (400 g of table salt in 1 L of distilled water) for flotation.

For soil samples, parasite egg recovery was carried out in accordance with the recommendations of (Horiuchi and Uga, 2016), who proposed a modified centrifugal flotation method.

Each fecal sample was analyzed an average of four times: twice using the McMaster flotation method and at least twice using the sedimentation technique. All examinations were conducted using a Leica DM750 microscope. A 10 × or 40 × objective was used to examine helminth eggs, while protozoa were assessed using the 40 × or 100 × objective. An integrated digital camera connected to a computer allowed for image capture and documentation. Parasites were identified based on morphological characteristics using standard identification keys (Garcia, 2001; Modrý et al., 2018; Zajac et al., 2012).

### 2.4 Statistical analyses

Statistical analyses were performed using R software, version 4.3.1. The prevalence of each parasite was calculated as the proportion of individuals testing positive for the parasite relative to the total number of individuals examined. Fisher's exact test was used to assess whether differences in parasite prevalence between groups (e.g., study sites) were statistically significant.

Binary logistic regression models were applied separately for each parasite taxon to assess the influence of intrinsic factors (sex, age class, and social status) on infection occurrence, as well as to explore associations between parasite presence and abiotic variables (soil surface temperature, soil water content, and precipitation). Only parasites exhibiting sufficient variability defined as the presence of both infected and uninfected individuals were included in the models. Odds ratios (OR) and their 90 % confidence intervals (CI) were calculated, with the significance threshold set at p < 0.05.

Finally, a Spearman rank correlation analysis was performed to examine the relationship between parasite prevalence in soil samples and in captive chimpanzees.

## 3 Results

### 3.1 Diversity, distribution, and prevalence of intestinal parasites identified in chimpanzees at the two study sites

**Fig. 2:**
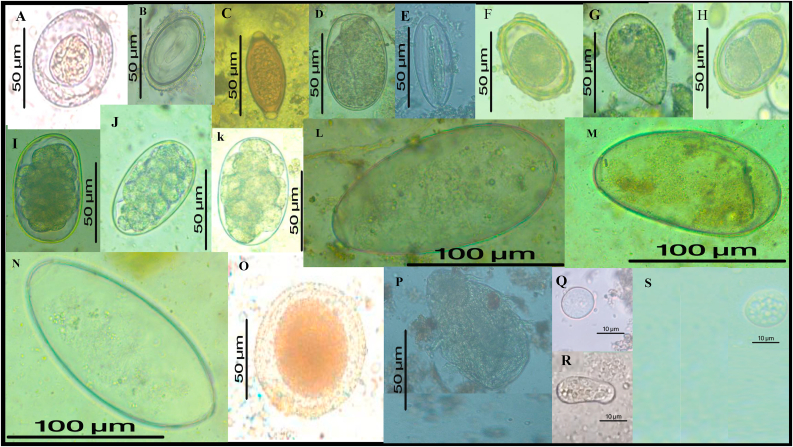
Diversity of intestinal parasites identified in chimpanzees. **Legend:***Hymenolepis* sp. (A); Spirurida fam. gen. (B); *Trichuris* sp*.* (C); *Strongyloides* sp. (D); *Enterobius* sp. (E); *Ascaris* sp. (F); *Balantioides coli.* (G); *Mamomonogamus* sp. (H); Strongylida fam. gen. (I, J, K); *Fasciola hepatica.* (L, M, N); *Toxocara* sp. (O); *Troglodytella* sp. (P); *Entamoeba* sp. (Q, R) *Endolimax nana* (S).

A total of 14 gastrointestinal parasite taxa were identified across the two study populations. These included four protozoa (*Entamoeba* sp., *Balantioides coli*, *Troglodytella* sp., and *Endolimax nana*) and ten helminths. The helminths comprised nematodes (*Trichuris* sp., *Ascaris* sp., *Strongyloides* sp., Strongylida fam. gen., *Mammomonogamus* sp., *Enterobius* sp., *Toxocara* sp., and Spirurida fam. gen.) and platyhelminths (*Hymenolepis* sp. and *Fasciola hepatica*). Notably, *Fasciola hepatica* and *Toxocara* sp. represent first reports in chimpanzees. (Fig. 2).

**Fig. 3:**
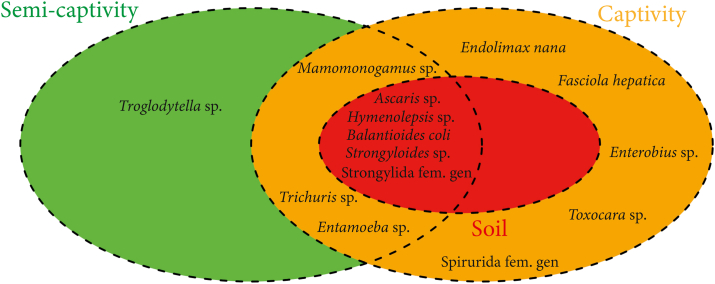
Distribution diagram of intestinal parasites among chimpanzees from Parc de la Lékédi, the Primate Center, and the soil.

In semi-captive individuals at Lékédi Park, nine parasitic taxa were detected, including *Trichuris* sp., *Entamoeba* sp., *Mammomonogamus* sp., *Ascaris* sp., *Balantioides coli*, *Hymenolepis* sp., *Strongyloides* sp., Strongylida fam. gen., and *Troglodytella* sp. In contrast, chimpanzees housed in strict captivity at the Primatology Center exhibited a broader parasitic profile, including all taxa listed above (except *Troglodytella* sp.) as well as *Endolimax nana*, *Fasciola hepatica*, *Enterobius* sp., *Toxocara* sp., and Spirurida fam. gen (Fig. 3).

Analysis of soil samples revealed five parasite taxa in common with host feces: *Ascaris* sp., *Balantioides coli*, *Hymenolepis* sp., *Strongyloides* sp., and Strongylida fam. gen., supporting environmental transmission pathways (Fig. 3).

The overall prevalence of gastrointestinal parasitic infections varied according to captivity status. Among chimpanzees kept in strict captivity, 85.4 % (35/41) were infected with at least one parasite. In contrast, the prevalence in semi-captive chimpanzees was higher, at 95.7 % (44/46).

The comparison of parasite prevalence between captive and semi-captive individuals revealed several statistically significant differences.

Among protozoan infections, *Balantioides coli* was significantly more prevalent in semi-captive chimpanzees (82.6 %) compared to those in strict captivity (22.0 %; *p* < 0.001). Similarly, infections with *Entamoeba* sp. (65.2 % vs. 39.0 %; *p* = 0.019) and *Troglodytella* sp. (21.7 % vs. 0 %; *p* = 0.001) were significantly more common in the semi-captive group.

Regarding helminth infections, *Strongyloides* sp. showed a notably higher prevalence in semi-captive chimpanzees compared to those in captivity (63.0 % vs. 17.1 %; *p* < 0.001). In addition, *Mammomonogamus* sp. was more frequently detected in semi-captive individuals (23.9 %) than in those under strict captivity (7.3 %; *p* = 0.044) (Table 2).

**Table 2:** Prevalence of intestinal parasites by site.

Taxa	Lékédi Park Prevalence (95 % CI)	Primatology Center Prevalence (95 % CI)	*p*-value Fisher's exact test
Protozoa			
*Entamoeba* sp.	30/46 (65.2 % [49.7–78.2])	16/41 (39.0 % [24.1–54.0])	0.019
*Endolimax nana*	0	3/41 (7.3 % [1.5–19.9])	0.1006
*Balantioides coli*	38/46 (82.6 % [68.6–92.2])	9/41 (22.0 % [10.6–37.6])	0.00001
*Troglodytella* sp.	10/46 (21.7 % [11.5–36.8])	0	0.0012
**Helminths**			
*Fasciola hepatica*	0	3/41 (7.3 % [0.0–15.3])	0.1
*Hymenolepis* sp.	2/46 (4.4 % [0.8–16.0])	2/41 (4.9 % [0.0–11.5])	1
*Mamomonogamus* sp.	11/46 (23.9 % [13.1–39.1])	3/41 (7.3 % [0.0–15.3])	0.0435
Spirurida fam. gen.	0	7/41 (17.1 % [5.6–28.6])	0.0038
Strongylida fam. gen.	40/46 (87.0 % [73.7–95.1])	29/41 (70.7 % [54.5–83.9])	0.071
*Strongyloides* sp.	29/46 (63.0 % [47.5–76.4])	7/41 (17.1 % [5.6–28.6])	0.0002
*Toxocara* sp.	0	1/41 (2.4 % [0.0–7.2])	0.47
*Trichuris* sp.	19/46 (41.3 % [27.3–56.7])	20/41 (48.8 % [33.5–64.1])	0.52
*Ascaris* sp.	9/46 (19.6 % [9.4–33.9])	9/41 (22.0 % [10.6–37.6])	0.797
*Enterobius* sp.	0	7/41 (17.1 % [7.2–32.1])	0.0038

Legend: The values "A/B (C% [D-E])" indicate, respectively, the number of infected animals (A), the total number examined (B), the prevalence (C%), and the corresponding 95 % confidence interval (D-E).

### 3.2 Influence of sex, age class, and social status on gastrointestinal parasite infections

**Fig. 4:**
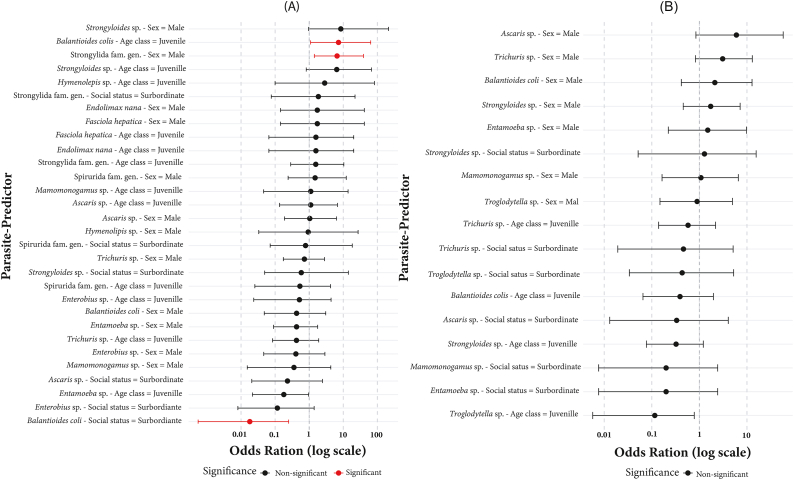
Forest plots showing the effects of intrinsic factors on gastrointestinal parasite occurrence in captive and semi-captive chimpanzees. Forest plots showing the effect of intrinsic factors (sex, age class, social status) on the occurrence of gastrointestinal parasites in captive (A) and semi-captive (B) chimpanzees. Each point represents an odds ratio (OR) with its 90 % confidence interval (CI). Odds ratios greater than 1 indicate an increased likelihood of infection associated with the predictor, whereas odds ratios less than 1 indicate a reduced likelihood. Red points correspond to statistically significant associations (p < 0.05), and black points correspond to non-significant associations.

In captive chimpanzees, Juvenile individuals exhibited a higher risk of infection with *Balantioides coli* compared to adults (OR = 7.24; 90 % CI: 2.15–24.3; p = 0.047). Social status was also significantly associated with *Balantioides coli* infection, with subordinate individuals being substantially less likely to be infected than dominant individuals (OR = 0.08; 90 % CI: 0.02–0.165; p = 0.007). Additionally, males had a significantly higher risk of infection with strongylid nematodes (Strongylida fam. gen.) compared to females (OR = 6.58; 90 % CI: 1.90–22.7; p = 0.023) (see Fig. 4A).In contrast, no significant associations were observed between sex, age class, or social status and the occurrence of parasitic infections among semi-captive individuals (Fig. 4B).

### 3.3 Relationship between abiotic variables and the occurrence of gastrointestinal parasites

Precipitation emerged as a particularly influential factor, being significantly associated with multiple parasites in both captive and semi-captive settings. In semi-captive individuals, higher precipitation levels were associated with an increased likelihood of infection with *Balantioides coli* (OR = 1.239, p = 0.0429), *Entamoeba* sp. (OR = 1.227, p = 0.0039), *Mammomonogamus* sp. (OR = 1.308, p = 0.0105), *Strongyloides* sp. (OR = 1.450, p = 0.0005), and *Trichuris* sp. (OR = 1.513, p = 0.0001). Conversely, in the captive population, *Entamoeba* sp. (OR = 0.788, p = 0.0179) and Strongylida fam. gen. (OR = 0.805, p = 0.0143) infections were negatively associated with precipitation, suggesting a protective effect. Soil surface temperature was also inversely correlated with the occurrence of *Balantioides coli* (OR = 0.034, p = 0.0429), *Entamoeba* sp. (OR = 0.040, p = 0.0039), and *Mammomonogamus* sp. (OR = 0.0147, p = 0.0105) in semi-captive chimpanzees. Importantly, no significant associations were detected between soil water content and parasite occurrence, indicating that this variable may have a limited role in shaping infection risks under the studied conditions (Table 3).

**Table 3:** Significant associations between climatic variables and the occurrence of gastrointestinal parasites in captive and semi-captive chimpanzees.

Site	Parasite	Climatic Variable	OR	90 % CI	p-value
Semi-captivity	*Balantioides coli*	Soil surface temperature (°C)	0.034	[0.0004–0.5441]	0.0429
Semi-captivity	*Balantioides coli*	Precipitation (mm/day)	1.239	[1.039–1.644]	0.0429
Captivity	*Entamoeba* sp.	Precipitation (mm/day)	0.788	[0.624–0.942]	0.0179
Semi-captivity	*Entamoeba* sp.	Soil surface temperature (°C)	0.040	[0.0034–0.307]	0.0039
Semi-captivity	*Entamoeba* sp.	Precipitation (mm/day)	1.227	[1.078–1.433]	0.0039
Semi-captivity	*Mammomonogamus* sp.	Soil surface temperature (°C)	0.0147	[0.0017–0.218]	0.0105
Semi-captivity	*Mammomonogamus* sp.	Precipitation (mm/day)	1.308	[1.102–1.732]	0.0105
Captivity	Strongylida fam. gen.	Precipitation (mm/day)	0.805	[0.669–0.952]	0.0143
Semi-captivity	*Strongyloides* sp.	Precipitation (mm/day)	1.450	[1.220–1.924]	0.0005
Semi-captivity	*Trichuris* sp.	Precipitation (mm/day)	1.513	[1.268–2.013]	0.0001

Legend: Only significant associations (p < 0.05) are reported. The table shows, for each parasite and climatic variable, the odds ratio (OR) with its corresponding 90 % confidence interval (CI) and p-value.

### 3.4 Relationship between soil contamination by intestinal parasites and chimpanzee infestation

**Fig. 5:**
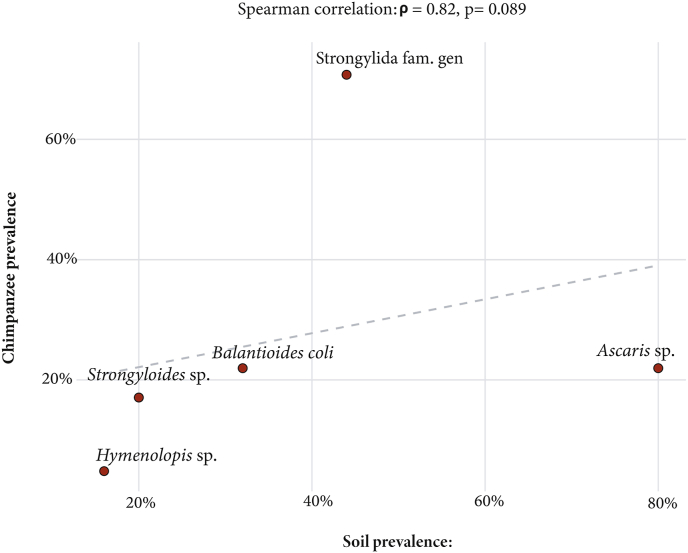
Relationship between soil infestation and chimpanzees' infestation.

The analysis of the relationship between soil contamination by intestinal parasites and chimpanzee infestation revealed a strong positive association (ρ = 0.82), although the result was not statistically significant (p = 0.089) (Fig. 5).

## 4 Discussion

This study highlights the combined influence of abiotic factors and individual characteristics on the emergence and spread of parasitic infections in captive and semi-captive chimpanzees.

It presents novel findings by documenting, for the first time, the presence of *Toxocara* sp. and *Fasciola hepatica* in chimpanzees. Except for these two parasites, all other taxa identified have already been reported in this host in both wild and captive contexts across various African countries (Akpan et al., 2010; Ashford et al., 2000; Birungi, 2022; Boundenga et al., 2021; Dibakou et al., 2021; Hasegawa et al., 2005; Howells et al., 2011; Hugot, 1993; Köster et al., 2021; Lilly et al., 2002; McLennan et al., 2017; Miller, 1968; Muehlenbein, 2005; Petrzelková et al., 2010). This high diversity, consistent with observations by Mbaya and Udendeye (2011), likely reflects a combination of high host density, repeated re-exposure through contaminated substrates, and the introduction of allochthonous parasites via diet or husbandry practices (Hasegawa and Udono, 2007; Vonfeld et al., 2022).

Two taxa warrant particular attention. *Fasciola hepatica*, a hepatobiliary trematode with a complex life cycle involving aquatic snails of the genus *Lymnaea*, is typically restricted to ruminants. Transmission is facilitated by proximity to aquatic environments (Fang et al., 2022; Gayo et al., 2020). Its detection in chimpanzees suggests a spill-over event from encysted metacercariae present in water or aquatic vegetation near the Mpassa River, located less than 10 m from the chimpanzees’ enrichment yard at the Primatology Center. In aberrant hosts, *F. hepatica* infections are typically subclinical but can impair liver function, emphasizing the need for targeted diagnostics and molluscicidal measures around stagnant water sources (CDC - DPDx - Fascioliasis, 2019). Likewise, the presence of *Toxocara* sp. most likely *T. canis* or *T. cati* in chimpanzees is attributable to environmental contamination by feces from guard dogs or pest control cats, which are the natural hosts of this parasite (Clark et al., 2014; Méndez et al., 2012; Warren, 1970). Although direct contact between these animals and chimpanzees is minimal, *Toxocara* eggs can persist in the soil for extended periods, increasing the likelihood of transmission. Fecal analyses of resident dogs confirmed the presence of *Toxocara* sp., raising zoonotic concerns for staff through the risk of visceral larva migrans (CDC - DPDx - Toxocariasis, 2019). Together, these findings highlight the permeability of captive systems to external parasitic pressures, challenging the notion that enclosure walls alone ensure sanitary isolation.

In terms of prevalence, overall infection rates reached 85.4 % in strictly captive chimpanzees and 95.7 % in semi-captive individuals figures exceeding those reported in earlier studies (Boundenga et al., 2021). However, the drivers of infection differed significantly. The forested enclosures of Lékédi Park favored soil- and waterborne parasites such as *Balantioides coli* and *Strongyloides* sp., whereas the concrete-floored enclosures of the Primatology Center hosted a broader, though less evenly distributed, community that included feed-borne and synanthropic taxa (*Enterobius*, *F. hepatica*, *Toxocara*) (A. F. White et al., 2019). Among them, *Balantioides coli* showed a fourfold higher prevalence in semi-captive chimpanzees (82.6 % vs. 22.0 %). This ciliate thrives in warm, humid environments and is efficiently transmitted through contaminated water or forage. Its rarity in strict captivity may result from routine enclosure cleaning and access to chlorinated drinking water, which disrupt the fecal oral cycle. Conversely, natural watercourses running through the forest enclosures at Lékédi likely maintain cyst viability. Similar ecological patterns whether favoring captivity or naturalistic conditionshave been observed in other African great ape sanctuaries, depending on water management strategies (Pomajbíková et al., 2010). These contrasts illustrate that “captivity” is not a uniform condition; rather, substrate types, drainage systems, and ecosystem connectivity determine which parasites proliferate.

Under strict captivity, individual host traits significantly modulated infection risk. Juvenile chimpanzees were approximately seven times more likely to harbor *B. coli* than adults, a pattern consistent with immature immune systems and exploratory behaviors typical of younger individuals, as seen in other captive New- and Old-World primates (da Silva Barbosa et al., 2015). Social hierarchy also played a key role: subordinate individuals were almost entirely spared from *B. coli* infection, likely because dominant individuals monopolize ground-level water and feeding areas where cysts tend to accumulate. This finding aligns with meta-analyses reporting higher parasite loads in dominant males across multiple primate species (Habig et al., 2019). Moreover, males carried six times more strongylid nematodes than females, reflecting well-documented patterns of androgen-mediated immunosuppression and increased exposure through territorial behaviors. None of these demographic effects were observed in semi-captive chimpanzees, suggesting that environmental heterogeneity and broader ranging in forest enclosures dilute individual-level risk factors (Klein, 2000, 2004).

Rainfall emerged as the principal abiotic driver, although its effects varied by management regime. In semi-captive settings, each increase in precipitation significantly raised the odds of infection for five taxa, notably *Strongyloides* sp. and *Trichuris* sp., reflecting these parasites’ strong dependence on moisture for the survival of their infective stages (A. F. White et al., 2019). By contrast, in concrete enclosures, heavy rainfall was associated with lower prevalence of *Entamoeba* and strongylid nematodes, likely due to efficient drainage that removes infective stages before they can be reencountered by the host. Additionally, soil surface temperature had a protective effect in semi-captive contexts: higher temperatures were negatively associated with *B. coli*, *Entamoeba*, and *Mammomonogamus* sp., consistent with thermal inactivation thresholds for these parasites (Schuster, 2003; Garcia, 2001).

The detection of five parasitic taxa (*Ascaris*, *Balantioides*, *Hymenolepis*, *Strongyloides*, Strongylida) in pen soils confirms the environmental seeding of infective stages and supports the plausibility of indirect, density-independent transmission loops. The strong Spearman correlation (ρ = 0.82) although not statistically significant between soil and fecal prevalence indicates a biologically relevant link. These findings are consistent with experimental studies showing that even low-level soil contamination can sustain endemic infections in primate groups through habitual geophagy or grooming behaviors (Hsu et al., 2002; Pebsworth et al., 2019).

## 5 Conclusions

In summary, this eco-epidemiological study reveals a remarkable diversity of gastrointestinal parasites, with 14 taxa identified in captive and semi-captive chimpanzees in Gabon, including the first records of *Fasciola hepatica* and *Toxocara* sp. in this species. While semi-captive individuals exhibited the highest overall prevalence, those housed under strict captivity showed greater taxonomic richness, suggesting that housing conditions strongly influence the composition of parasite communities. In captivity, intrinsic factors such as age, sex, and social status significantly shaped infection risk, whereas in semi-captivity, abiotic variables particularly precipitation and soil temperature were the main drivers. The strong correlation observed between substrate contamination and host infestation further underscores the central role of soil as a persistent reservoir sustaining reinfection cycles.

These findings call for a differentiated health management strategy, combining environmental interventions (e.g., drainage systems, rotational use of foraging areas, substrate sanitation) with integrated surveillance under a One Health framework. Such an approach is essential to mitigate parasitic pressure and prevent zoonotic transmission in both types of captive settings.

Future research should incorporate high-sensitivity diagnostic tools, such as quantitative PCR, high-resolution microclimatic monitoring, and longitudinal sampling. These methodologies will improve our understanding of parasitic dynamics, clarify transmission pathways, and enhance preparedness against emerging health risks.

## CRediT authorship contribution statement

**Mohamed Hassani Mohamed-Djawad:** Writing – original draft, Software, Methodology, Investigation, Formal analysis, Data curation, Conceptualization. **Krista Mapagha-Boundoukou:** Writing – original draft, Investigation, Formal analysis, Data curation. **Neil M. Longo-Pendy:** Writing – review & editing, Software, Resources, Formal analysis. **Serge Ely Dibakou:** Writing – review & editing, Methodology, Data curation. **Barthelemy Ngoubangoye:** Writing – review & editing, Validation, Software, Investigation, Conceptualization. **Papa Ibnou Ndiaye:** Writing – review & editing, Validation, Supervision, Conceptualization. **Larson Boundenga:** Writing – review & editing, Visualization, Validation, Supervision, Resources, Project administration, Funding acquisition, Data curation.

## Institutional review board statement:

The study was approved by the National Ethics Committee of Gabon and authorized by the Gabonese Ministries of Water and Forests, Higher Education, Scientific Research, and Innovation (Approval No. AR0031/10/MENESRESI/CENAREST/CG/CST/CSAR).

## Funding

The study was funded by the Centre International de Recherches Médicales de Franceville (10.13039/501100004466CIRMF, Gabon). We thank all the persons involved in the sampling. We also thank Mohamed Ibrahim for her help with English editing.

## Conflict of interest

The authors declare that they have no conflicts of interest related to this study.
